# 2-Hy­droxy-*N*,*N*,*N*-trimethyl-3-tetra­decyl­oxypropan-1-aminium bromide

**DOI:** 10.1107/S1600536811054651

**Published:** 2011-12-23

**Authors:** Xiuhong Wang, Xilian Wei, Panpan Du

**Affiliations:** a College of Chemistry and Chemical Engineering, Liaocheng University, Shandong 252059, People’s Republic of China

## Abstract

In the crystal structure of the title compound, C_20_H_44_NO_2_^+^·Br^−^, the cation and anion are connected *via* an O—H⋯Br hydrogen bond, forming an ionic pair. The cation is disordered over two conformations related by a mirror plane, and the anion is situated on a mirror plane so that the asymmetric unit contains half of the ionic pair. The long alkyl chain in the cation adopts an all-*trans* conformation. The crystal packing exhibits weak inter­molecular C—H⋯O inter­actions.

## Related literature

For related structures, see: Koh *et al.* (1993 ▶); Fu *et al.* (2009 ▶); Liu *et al.* (2010 ▶). For details of the synthesis, see: Yin *et al.* (1998 ▶).
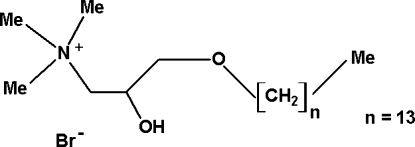


## Experimental

### 

#### Crystal data

C_20_H_44_NO_2_^+^·Br^−^
*M* *_r_* = 410.47
Monoclinic, 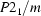

*a* = 5.9470 (4) Å
*b* = 7.4331 (5) Å
*c* = 26.720 (2) Å
β = 92.185 (1)°
*V* = 1180.30 (15) Å^3^
*Z* = 2
Mo *K*α radiation
μ = 1.75 mm^−1^
*T* = 293 K
0.39 × 0.32 × 0.30 mm

#### Data collection

Bruker SMART CCD area-detector diffractometer
Absorption correction: multi-scan (*SADABS*; Sheldrick, 1996 ▶) *T*_min_ = 0.548, *T*_max_ = 0.621
5657 measured reflections
2251 independent reflections
1430 reflections with *I* > 2σ(*I*)
*R*_int_ = 0.048

#### Refinement

*R*[*F* ^2^ > 2σ(*F* ^2^)] = 0.052
*wR*(*F* ^2^) = 0.154
*S* = 0.99
2251 reflections
171 parameters
H-atom parameters constrained
Δρ_max_ = 0.36 e Å^−3^
Δρ_min_ = −0.42 e Å^−3^

### 

Data collection: *SMART* (Bruker, 2007 ▶); cell refinement: *SAINT* (Bruker, 2007 ▶); data reduction: *SAINT*; program(s) used to solve structure: *SHELXS97* (Sheldrick, 2008 ▶); program(s) used to refine structure: *SHELXL97* (Sheldrick, 2008 ▶); molecular graphics: *SHELXTL* (Sheldrick, 2008 ▶); software used to prepare material for publication: *SHELXTL*.

## Supplementary Material

Crystal structure: contains datablock(s) I, global. DOI: 10.1107/S1600536811054651/cv5198sup1.cif

Structure factors: contains datablock(s) I. DOI: 10.1107/S1600536811054651/cv5198Isup2.hkl

Supplementary material file. DOI: 10.1107/S1600536811054651/cv5198Isup3.cml

Additional supplementary materials:  crystallographic information; 3D view; checkCIF report

## Figures and Tables

**Table 1:** Hydrogen-bond geometry (Å, °)

*D*—H⋯*A*	*D*—H	H⋯*A*	*D*⋯*A*	*D*—H⋯*A*
O1—H1*D*⋯Br1	0.85	2.41	3.207 (6)	157
C19—H19*B*⋯O1^i^	0.96	2.23	3.190 (6)	176

Symmetry code: (i) 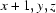.

**Fig. 1.**
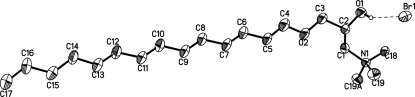
The molecular structure of the title compound, with atom labeling scheme and 50% probability displacement ellipsoids [symmetry code: (A) x, 1/2-y, z]. Only one conformation of the cation is shown. Dashed line denotes hydrogen bond. C-bound H-atoms omitted for clarity.

## supplementary crystallographic information

### Comment

Cationic surfactants have various applications serving as fabric softeners, disinfectants, demulsifiers, emulsifiers, wetting agents and processing aids. Quaternary ammonium based surfactants are molecules with at least one hydrophobic long alkyl chain attached to a positively charged nitrogen atom. Synthesis of 3-alkoxy-2-hydroxypropyl-*N*,*N*,*N*-trimethylpropan- 1-aminium bromides (RTABs) was described by Yin *et al.* (1998). As a part of the studies on the chemistry of surfactants, we report here the crystal structure of the title compound (I).

In (I) (Fig. 1), all bond lengths and angles are normal and correspond to those observed in the related compounds (Koh *et al.*, 1993; Fu *et al.*, 2009; Liu *et al.*, 2010). The C3—O2 and C4—O2 bond lengths are 1.411 (10) and 1.373 (7) Å, respectively. The cation and anion  are connected by O—H···Br hydrogen bond (Table 1) forming an ionic pair. The cation is disordered over two conformations related by the mirror plane, and anion is situated on a mirror plane so asymmetric unit contains a half of the ionic pair.

The crystal packing exhibits weak intermolecular C—H···O interactions (Table 1).

### Experimental

The reaction was carried out under nitrogen atmosphere. Trimethylammonium bromide (0.12 mol) and tetradecyl glcidyl ether (0.1 mol) were added to a stirred solution of ethanol (100 ml) and stirred at 320 K for 24 h. The resulting clear solution was evaporated under vacuum. Colorless crystals suitable for X-ray analysis were obtained by slow evaporation of a ethyl acetate solution over a period of two weeks. (yield 78%,m.p. 342 K) Anal. Calcd(%) for C_20_H_44_NO_2_^+^Br^-^ (410.47): C, 57.87; H, 10.72; N, 3.41. Found (%): C, 58.48, H, 10.82 N, 3.48.

### Refinement

All H atoms were placed geometrically and treated as riding on their parent atoms with O—H distances of 0.85 Å, C—H distances of 0.97 Å (methylene), C—H distances of 0.96 Å (methyl). The*U*_iso_(H) values were set at 1.2*U*_eq_ for the methylene H atoms and at 1.5*U*_eq_ for other H atoms.

### Crystal data

**Table:**

C_20_H_44_NO_2_^+^·Br^−^	*F*(000) = 444
*M**_r_* = 410.47	*D*_x_ = 1.155 Mg m^−^^3^
Monoclinic, *P*2_1_/*m*	Mo *K*α radiation, λ = 0.71073 Å
*a* = 5.9470 (4) Å	Cell parameters from 1899 reflections
*b* = 7.4331 (5) Å	θ = 2.7–20.7°
*c* = 26.720 (2) Å	µ = 1.75 mm^−^^1^
β = 92.185 (1)°	*T* = 293 K
*V* = 1180.30 (15) Å^3^	Block, colourless
*Z* = 2	0.39 × 0.32 × 0.30 mm

### Data collection

**Table:**

Bruker SMART CCD area-detector diffractometer	2251 independent reflections
Radiation source: fine-focus sealed tube	1430 reflections with *I* > 2σ(*I*)
graphite	*R*_int_ = 0.048
phi and ω scans	θ_max_ = 25.0°, θ_min_ = 2.8°
Absorption correction: multi-scan (*SADABS*; Sheldrick, 1996)	*h* = −7→6
*T*_min_ = 0.548, *T*_max_ = 0.621	*k* = −6→8
5657 measured reflections	*l* = −30→31

### Refinement

**Table:**

Refinement on *F*^2^	Primary atom site location: structure-invariant direct methods
Least-squares matrix: full	Secondary atom site location: difference Fourier map
*R*[*F*^2^ > 2σ(*F*^2^)] = 0.052	Hydrogen site location: inferred from neighbouring sites
*wR*(*F*^2^) = 0.154	H-atom parameters constrained
*S* = 0.99	*w* = 1/[σ^2^(*F*_o_^2^) + (0.0907*P*)^2^] where *P* = (*F*_o_^2^ + 2*F*_c_^2^)/3
2251 reflections	(Δ/σ)_max_ = 0.001
171 parameters	Δρ_max_ = 0.36 e Å^−^^3^
0 restraints	Δρ_min_ = −0.42 e Å^−^^3^

### Special details

**Table:**

Geometry. All e.s.d.'s (except the e.s.d. in the dihedral angle between two l.s. planes) are estimated using the full covariance matrix. The cell e.s.d.'s are taken into account individually in the estimation of e.s.d.'s in distances, angles and torsion angles; correlations between e.s.d.'s in cell parameters are only used when they are defined by crystal symmetry. An approximate (isotropic) treatment of cell e.s.d.'s is used for estimating e.s.d.'s involving l.s. planes.
Refinement. Refinement of *F*^2^ against ALL reflections. The weighted *R*-factor *wR* and goodness of fit *S* are based on *F*^2^, conventional *R*-factors *R* are based on *F*, with *F* set to zero for negative *F*^2^. The threshold expression of *F*^2^ > σ(*F*^2^) is used only for calculating *R*-factors(gt) *etc*. and is not relevant to the choice of reflections for refinement. *R*-factors based on *F*^2^ are statistically about twice as large as those based on *F*, and *R*- factors based on ALL data will be even larger.

### Fractional atomic coordinates and isotropic or equivalent isotropic displacement parameters (Å^2^)

**Table:**

	*x*	*y*	*z*	*U*_iso_*/*U*_eq_	Occ. (<1)
Br1	−0.29860 (11)	0.7500	0.07517 (2)	0.0781 (3)	
O1	−0.3202 (8)	0.3776 (8)	0.13541 (18)	0.0719 (15)	0.50
H1D	−0.2738	0.4655	0.1183	0.086*	0.50
O2	0.0819 (8)	0.2500	0.23481 (14)	0.1043 (17)	
N1	0.0902 (7)	0.2500	0.07672 (15)	0.0570 (11)	
C1	0.0206 (14)	0.1957 (11)	0.1295 (3)	0.070 (3)	0.50
H1A	−0.0773	0.0914	0.1269	0.084*	0.50
H1B	0.1538	0.1618	0.1494	0.084*	0.50
C2	−0.1016 (13)	0.3479 (12)	0.1561 (3)	0.064 (2)	0.50
H2	−0.0133	0.4590	0.1549	0.077*	0.50
C3	−0.1261 (14)	0.290 (4)	0.2101 (2)	0.087 (8)	0.50
H3A	−0.1997	0.3853	0.2282	0.104*	0.50
H3B	−0.2218	0.1843	0.2108	0.104*	0.50
C4	0.0878 (14)	0.2500	0.2862 (2)	0.113 (3)	
H4A	0.0049	0.1452	0.2968	0.135*	0.50
H4B	0.0049	0.3548	0.2968	0.135*	0.50
C5	0.2932 (11)	0.2500	0.3127 (2)	0.091 (2)	
H5A	0.3553	0.3698	0.3092	0.109*	0.50
H5B	0.3907	0.1694	0.2950	0.109*	0.50
C6	0.3204 (15)	0.203 (3)	0.3659 (3)	0.096 (8)	0.50
H6A	0.1945	0.2500	0.3826	0.116*	
H6B	0.3032	0.0739	0.3683	0.116*	0.50
C7	0.5191 (12)	0.2500	0.3947 (2)	0.090 (2)	
H7A	0.5322	0.3800	0.3935	0.108*	0.50
H7B	0.6454	0.2018	0.3771	0.108*	0.50
C8	0.5521 (14)	0.1965 (14)	0.4480 (3)	0.086 (4)	0.50
H8A	0.4234	0.2500	0.4655	0.103*	
H8B	0.5452	0.0662	0.4492	0.103*	0.50
C9	0.7504 (12)	0.2500	0.4770 (2)	0.087 (2)	
H9A	0.7576	0.3803	0.4760	0.104*	0.50
H9B	0.8796	0.2057	0.4596	0.104*	0.50
C10	0.7807 (16)	0.1948 (14)	0.5305 (3)	0.089 (4)	0.50
H10A	0.6513	0.2500	0.5479	0.107*	
H10B	0.7751	0.0645	0.5315	0.107*	0.50
C11	0.9785 (12)	0.2500	0.5590 (2)	0.090 (2)	
H11A	0.9839	0.3803	0.5577	0.108*	0.50
H11B	1.1074	0.2063	0.5414	0.108*	0.50
C12	1.0140 (15)	0.1975 (15)	0.6127 (3)	0.089 (5)	0.50
H12A	0.8865	0.2500	0.6304	0.107*	
H12B	1.0059	0.0672	0.6141	0.107*	0.50
C13	1.2124 (13)	0.2500	0.6409 (2)	0.096 (2)	
H13A	1.2690	0.3530	0.6229	0.116*	0.50
H13B	1.3187	0.1536	0.6355	0.116*	0.50
C14	1.2474 (15)	0.297 (2)	0.6938 (3)	0.098 (7)	0.50
H14A	1.2309	0.4267	0.6963	0.118*	0.50
H14B	1.1238	0.2500	0.7115	0.118*	
C15	1.4523 (14)	0.2500	0.7218 (2)	0.112 (3)	
H15A	1.4903	0.1295	0.7110	0.135*	0.50
H15B	1.5678	0.3286	0.7094	0.135*	0.50
C16	1.4849 (15)	0.2500	0.7756 (3)	0.130 (3)	
H16A	1.4050	0.3544	0.7875	0.155*	0.50
H16B	1.4050	0.1456	0.7875	0.155*	0.50
C17	1.6923 (17)	0.2500	0.8012 (3)	0.157 (4)	
H17A	1.6703	0.2500	0.8366	0.235*	
H17B	1.7751	0.3555	0.7923	0.235*	0.50
H17C	1.7751	0.1445	0.7923	0.235*	0.50
C18	−0.1067 (9)	0.2500	0.04103 (18)	0.0646 (15)	
H18A	−0.1988	0.3532	0.0471	0.097*	0.50
H18B	−0.1930	0.1424	0.0455	0.097*	0.50
H18C	−0.0558	0.2544	0.0074	0.097*	0.50
C19	0.2289 (7)	0.4126 (7)	0.06755 (16)	0.0844 (13)	
H19A	0.2678	0.4158	0.0330	0.127*	
H19B	0.3638	0.4084	0.0885	0.127*	
H19C	0.1447	0.5186	0.0753	0.127*	

### Atomic displacement parameters (Å^2^)

**Table:**

	*U*^11^	*U*^22^	*U*^33^	*U*^12^	*U*^13^	*U*^23^
Br1	0.0846 (5)	0.0628 (4)	0.0888 (5)	0.000	0.0271 (3)	0.000
O1	0.060 (3)	0.087 (4)	0.069 (3)	0.000 (3)	0.000 (3)	−0.004 (3)
O2	0.089 (3)	0.183 (5)	0.041 (2)	0.000	0.005 (2)	0.000
N1	0.047 (2)	0.074 (3)	0.050 (3)	0.000	0.0014 (19)	0.000
C1	0.071 (5)	0.088 (10)	0.052 (4)	0.015 (4)	−0.003 (4)	0.002 (4)
C2	0.059 (5)	0.087 (5)	0.045 (5)	0.008 (4)	0.003 (4)	−0.002 (4)
C3	0.082 (5)	0.13 (3)	0.052 (4)	−0.001 (6)	0.008 (4)	0.006 (6)
C4	0.108 (6)	0.173 (9)	0.058 (5)	0.000	0.007 (4)	0.000
C5	0.095 (5)	0.139 (6)	0.039 (3)	0.000	0.003 (3)	0.000
C6	0.103 (6)	0.14 (2)	0.047 (4)	0.009 (7)	0.000 (4)	0.000 (5)
C7	0.104 (5)	0.125 (6)	0.042 (4)	0.000	0.009 (3)	0.000
C8	0.100 (6)	0.109 (13)	0.047 (4)	0.000 (5)	0.001 (4)	0.000 (4)
C9	0.105 (5)	0.112 (6)	0.044 (3)	0.000	0.008 (3)	0.000
C10	0.107 (6)	0.114 (13)	0.047 (4)	0.005 (5)	−0.001 (4)	−0.002 (4)
C11	0.103 (5)	0.122 (6)	0.046 (4)	0.000	0.007 (3)	0.000
C12	0.110 (6)	0.110 (14)	0.047 (4)	−0.003 (6)	−0.003 (4)	−0.005 (4)
C13	0.115 (6)	0.127 (6)	0.048 (4)	0.000	0.007 (4)	0.000
C14	0.117 (7)	0.13 (2)	0.043 (4)	0.025 (8)	−0.002 (4)	0.000 (5)
C15	0.113 (6)	0.169 (9)	0.055 (4)	0.000	0.010 (4)	0.000
C16	0.137 (8)	0.197 (10)	0.053 (5)	0.000	−0.017 (5)	0.000
C17	0.132 (8)	0.273 (15)	0.065 (5)	0.000	0.000 (5)	0.000
C18	0.064 (3)	0.087 (4)	0.042 (3)	0.000	0.000 (3)	0.000
C19	0.079 (3)	0.081 (3)	0.094 (3)	−0.015 (2)	0.004 (2)	−0.007 (2)

### Geometric parameters (Å, °)

**Table:**

O1—C2	1.411 (8)	C9—C8^i^	1.441 (9)
O1—H1D	0.8500	C9—C10	1.493 (9)
O2—C4	1.373 (7)	C9—C10^i^	1.493 (9)
O2—C3^i^	1.411 (10)	C9—H9A	0.9700
O2—C3	1.411 (10)	C9—H9B	0.9700
N1—C18	1.482 (6)	C10—C10^i^	0.82 (2)
N1—C19	1.489 (5)	C10—C11	1.437 (10)
N1—C19^i^	1.489 (5)	C10—H10A	1.0021
N1—C1	1.538 (8)	C10—H10B	0.9700
N1—C1^i^	1.538 (8)	C11—C10^i^	1.437 (10)
C1—C1^i^	0.808 (16)	C11—C12	1.493 (9)
C1—C2^i^	1.087 (9)	C11—C12^i^	1.493 (9)
C1—C2	1.535 (10)	C11—H11A	0.9700
C1—H1A	0.9700	C11—H11B	0.9701
C1—H1B	0.9699	C12—C12^i^	0.78 (2)
C2—C1^i^	1.087 (9)	C12—C13	1.430 (10)
C2—C2^i^	1.455 (17)	C12—H12A	0.9892
C2—C3	1.518 (12)	C12—H12B	0.9700
C2—C3^i^	1.780 (19)	C13—C12^i^	1.430 (10)
C2—H2	0.9800	C13—C14	1.465 (10)
C3—C3^i^	0.59 (6)	C13—C14^i^	1.465 (10)
C3—C2^i^	1.780 (19)	C13—H13A	0.9700
C3—H3A	0.9700	C13—H13B	0.9700
C3—H3B	0.9700	C14—C14^i^	0.70 (4)
C4—C5	1.389 (9)	C14—C15	1.448 (11)
C4—H4A	0.9700	C14—H14A	0.9701
C4—H4B	0.9700	C14—H14B	0.9543
C5—C6	1.465 (10)	C15—C16	1.443 (9)
C5—C6^i^	1.465 (10)	C15—C14^i^	1.448 (11)
C5—H5A	0.9700	C15—H15A	0.9701
C5—H5B	0.9700	C15—H15B	0.9701
C6—C6^i^	0.69 (4)	C16—C17	1.387 (11)
C6—C7	1.428 (10)	C16—H16A	0.9700
C6—H6A	0.9514	C16—H16B	0.9700
C6—H6B	0.9699	C17—H17A	0.9600
C7—C6^i^	1.428 (10)	C17—H17B	0.9600
C7—C8	1.486 (8)	C17—H17C	0.9600
C7—C8^i^	1.486 (8)	C18—H18A	0.9600
C7—H7A	0.9700	C18—H18B	0.9600
C7—H7B	0.9698	C18—H18C	0.9600
C8—C8^i^	0.80 (2)	C19—H19A	0.9600
C8—C9	1.441 (9)	C19—H19B	0.9600
C8—H8A	0.9952	C19—H19C	0.9600
C8—H8B	0.9700		
			
C2—O1—H1D	91.1	C9—C8—H8A	105.5
C4—O2—C3^i^	117.1 (6)	C7—C8—H8A	105.5
C4—O2—C3	117.1 (6)	C8^i^—C8—H8B	176.9
C3^i^—O2—C3	24 (2)	C9—C8—H8B	107.1
C18—N1—C19	108.9 (3)	C7—C8—H8B	107.1
C18—N1—C19^i^	108.9 (3)	H8A—C8—H8B	110.4
C19—N1—C19^i^	108.5 (5)	C8^i^—C9—C8	32.0 (8)
C18—N1—C1	110.9 (4)	C8^i^—C9—C10	131.0 (7)
C19—N1—C1	122.3 (4)	C8—C9—C10	120.3 (6)
C19^i^—N1—C1	96.2 (4)	C8^i^—C9—C10^i^	120.3 (6)
C18—N1—C1^i^	110.9 (4)	C8—C9—C10^i^	131.0 (7)
C19—N1—C1^i^	96.2 (4)	C10—C9—C10^i^	31.9 (8)
C19^i^—N1—C1^i^	122.3 (4)	C8^i^—C9—H9A	75.3
C1—N1—C1^i^	30.5 (6)	C8—C9—H9A	107.3
C18—N1—Br1	65.78 (10)	C10—C9—H9A	107.2
C19—N1—Br1	66.7 (2)	C10^i^—C9—H9A	75.4
C19^i^—N1—Br1	169.6 (3)	C8^i^—C9—H9B	119.0
C1—N1—Br1	94.1 (3)	C8—C9—H9B	107.3
C1^i^—N1—Br1	68.0 (3)	C10—C9—H9B	107.2
C1^i^—C1—C2^i^	107.3 (6)	C10^i^—C9—H9B	118.9
C1^i^—C1—C2	42.5 (4)	H9A—C9—H9B	106.9
C2^i^—C1—C2	64.8 (8)	C10^i^—C10—C11	73.4 (4)
C1^i^—C1—N1	74.8 (3)	C10^i^—C10—C9	74.0 (4)
C2^i^—C1—N1	152.8 (8)	C11—C10—C9	119.8 (7)
C2—C1—N1	112.4 (6)	C10^i^—C10—H10A	65.8
C1^i^—C1—H1A	143.0	C11—C10—H10A	105.3
C2^i^—C1—H1A	52.6	C9—C10—H10A	105.4
C2—C1—H1A	109.1	C10^i^—C10—H10B	177.4
N1—C1—H1A	109.1	C11—C10—H10B	107.4
C1^i^—C1—H1B	105.0	C9—C10—H10B	107.4
C2^i^—C1—H1B	96.7	H10A—C10—H10B	111.6
C2—C1—H1B	109.1	C10^i^—C11—C10	33.2 (9)
N1—C1—H1B	109.1	C10^i^—C11—C12	131.8 (7)
H1A—C1—H1B	107.9	C10—C11—C12	121.2 (6)
C1^i^—C2—O1	114.8 (7)	C10^i^—C11—C12^i^	121.2 (6)
C1^i^—C2—C2^i^	72.7 (6)	C10—C11—C12^i^	131.8 (7)
O1—C2—C2^i^	99.0 (4)	C12—C11—C12^i^	30.3 (8)
C1^i^—C2—C3	129.0 (11)	C10^i^—C11—H11A	73.9
O1—C2—C3	107.3 (6)	C10—C11—H11A	107.0
C2^i^—C2—C3	73.5 (11)	C12—C11—H11A	107.0
C1^i^—C2—C1	30.2 (7)	C12^i^—C11—H11A	76.7
O1—C2—C1	112.3 (6)	C10^i^—C11—H11B	118.9
C2^i^—C2—C1	42.5 (4)	C10—C11—H11B	107.0
C3—C2—C1	107.3 (11)	C12—C11—H11B	107.0
C1^i^—C2—C3^i^	116.0 (9)	C12^i^—C11—H11B	117.9
O1—C2—C3^i^	107.8 (6)	H11A—C11—H11B	106.8
C2^i^—C2—C3^i^	54.9 (8)	C12^i^—C12—C13	74.1 (4)
C3—C2—C3^i^	18.7 (18)	C12^i^—C12—C11	74.8 (4)
C1—C2—C3^i^	90.4 (8)	C13—C12—C11	121.2 (7)
C1^i^—C2—H2	81.7	C12^i^—C12—H12A	66.7
O1—C2—H2	110.0	C13—C12—H12A	105.8
C2^i^—C2—H2	147.4	C11—C12—H12A	105.8
C3—C2—H2	110.0	C12^i^—C12—H12B	176.3
C1—C2—H2	110.0	C13—C12—H12B	107.1
C3^i^—C2—H2	124.9	C11—C12—H12B	107.0
C3^i^—C3—O2	77.9 (11)	H12A—C12—H12B	109.6
C3^i^—C3—C2	106.5 (11)	C12^i^—C13—C12	31.7 (9)
O2—C3—C2	112.9 (6)	C12^i^—C13—C14	121.8 (7)
C3^i^—C3—C2^i^	54.9 (8)	C12—C13—C14	131.1 (7)
O2—C3—C2^i^	99.2 (11)	C12^i^—C13—C14^i^	131.1 (7)
C2—C3—C2^i^	51.6 (7)	C12—C13—C14^i^	121.8 (7)
C3^i^—C3—H3A	136.9	C14—C13—C14^i^	27.7 (14)
O2—C3—H3A	109.0	C12^i^—C13—H13A	79.6
C2—C3—H3A	109.0	C12—C13—H13A	104.5
C2^i^—C3—H3A	151.0	C14—C13—H13A	104.4
C3^i^—C3—H3B	36.0	C14^i^—C13—H13A	128.8
O2—C3—H3B	109.0	C12^i^—C13—H13B	130.8
C2—C3—H3B	109.0	C12—C13—H13B	104.5
C2^i^—C3—H3B	67.4	C14—C13—H13B	104.5
H3A—C3—H3B	107.8	C14^i^—C13—H13B	84.1
O2—C4—C5	119.9 (6)	H13A—C13—H13B	105.6
O2—C4—H4A	107.3	C14^i^—C14—C15	76.0 (7)
C5—C4—H4A	107.3	C14^i^—C14—C13	76.2 (7)
O2—C4—H4B	107.3	C15—C14—C13	121.8 (9)
C5—C4—H4B	107.3	C14^i^—C14—H14A	172.9
H4A—C4—H4B	106.9	C15—C14—H14A	106.9
C4—C5—C6	123.9 (7)	C13—C14—H14A	106.9
C4—C5—C6^i^	123.9 (6)	C14^i^—C14—H14B	68.5
C6—C5—C6^i^	27.4 (16)	C15—C14—H14B	107.8
C4—C5—H5A	106.4	C13—C14—H14B	107.8
C6—C5—H5A	106.4	H14A—C14—H14B	104.4
C6^i^—C5—H5A	81.3	C16—C15—C14^i^	126.3 (7)
C4—C5—H5B	106.3	C16—C15—C14	126.3 (7)
C6—C5—H5B	106.3	C14^i^—C15—C14	28.0 (14)
C6^i^—C5—H5B	125.0	C16—C15—H15A	105.7
H5A—C5—H5B	106.4	C14^i^—C15—H15A	79.9
C6^i^—C6—C7	75.9 (8)	C14—C15—H15A	105.8
C6^i^—C6—C5	76.3 (8)	C16—C15—H15B	105.7
C7—C6—C5	121.6 (9)	C14^i^—C15—H15B	124.2
C6^i^—C6—H6A	68.6	C14—C15—H15B	105.7
C7—C6—H6A	108.0	H15A—C15—H15B	106.2
C5—C6—H6A	108.0	C17—C16—C15	125.0 (8)
C6^i^—C6—H6B	172.7	C17—C16—H16A	106.1
C7—C6—H6B	107.0	C15—C16—H16A	106.1
C5—C6—H6B	107.0	C17—C16—H16B	106.1
H6A—C6—H6B	104.1	C15—C16—H16B	106.1
C6^i^—C7—C6	28.1 (16)	H16A—C16—H16B	106.3
C6^i^—C7—C8	131.2 (7)	C16—C17—H17A	109.5
C6—C7—C8	121.9 (7)	C16—C17—H17B	109.5
C6^i^—C7—C8^i^	121.9 (7)	H17A—C17—H17B	109.5
C6—C7—C8^i^	131.2 (7)	C16—C17—H17C	109.5
C8—C7—C8^i^	31.0 (8)	H17A—C17—H17C	109.5
C6^i^—C7—H7A	78.9	H17B—C17—H17C	109.5
C6—C7—H7A	106.9	N1—C18—H18A	109.5
C8—C7—H7A	106.8	N1—C18—H18B	109.5
C8^i^—C7—H7A	75.9	H18A—C18—H18B	109.5
C6^i^—C7—H7B	118.0	N1—C18—H18C	109.5
C6—C7—H7B	106.8	H18A—C18—H18C	109.5
C8—C7—H7B	106.8	H18B—C18—H18C	109.5
C8^i^—C7—H7B	119.2	N1—C19—H19A	109.5
H7A—C7—H7B	106.7	N1—C19—H19B	109.5
C8^i^—C8—C9	74.0 (4)	H19A—C19—H19B	109.5
C8^i^—C8—C7	74.5 (4)	N1—C19—H19C	109.5
C9—C8—C7	121.1 (7)	H19A—C19—H19C	109.5
C8^i^—C8—H8A	66.5	H19B—C19—H19C	109.5
			
C18—N1—C1—C1^i^	−95.96 (18)	C4—C5—C6—C6^i^	−99.4 (6)
C19—N1—C1—C1^i^	34.6 (3)	C4—C5—C6—C7	−163.1 (8)
C19^i^—N1—C1—C1^i^	151.1 (3)	C6^i^—C5—C6—C7	−63.7 (13)
Br1—N1—C1—C1^i^	−30.35 (7)	C5—C6—C7—C6^i^	63.9 (13)
C18—N1—C1—C2^i^	2.4 (19)	C6^i^—C6—C7—C8	118.7 (7)
C19—N1—C1—C2^i^	132.9 (17)	C5—C6—C7—C8	−177.4 (10)
C19^i^—N1—C1—C2^i^	−110.5 (18)	C6^i^—C6—C7—C8^i^	81.5 (9)
C1^i^—N1—C1—C2^i^	98.3 (18)	C5—C6—C7—C8^i^	145.4 (9)
Br1—N1—C1—C2^i^	68.0 (18)	C6^i^—C7—C8—C8^i^	−84.7 (11)
C18—N1—C1—C2	−75.7 (6)	C6—C7—C8—C8^i^	−118.0 (10)
C19—N1—C1—C2	54.9 (8)	C6^i^—C7—C8—C9	−144.6 (12)
C19^i^—N1—C1—C2	171.4 (6)	C6—C7—C8—C9	−177.9 (10)
C1^i^—N1—C1—C2	20.3 (6)	C8^i^—C7—C8—C9	−59.9 (8)
Br1—N1—C1—C2	−10.1 (6)	C7—C8—C9—C8^i^	60.1 (8)
C2^i^—C1—C2—C1^i^	180.000 (4)	C8^i^—C8—C9—C10	119.9 (6)
N1—C1—C2—C1^i^	−29.6 (9)	C7—C8—C9—C10	−179.9 (7)
C1^i^—C1—C2—O1	101.3 (7)	C8^i^—C8—C9—C10^i^	82.6 (6)
C2^i^—C1—C2—O1	−78.7 (7)	C7—C8—C9—C10^i^	142.7 (7)
N1—C1—C2—O1	71.7 (8)	C8^i^—C9—C10—C10^i^	−82.4 (6)
C1^i^—C1—C2—C2^i^	180.000 (3)	C8—C9—C10—C10^i^	−120.0 (7)
N1—C1—C2—C2^i^	150.4 (9)	C8^i^—C9—C10—C11	−142.0 (8)
C1^i^—C1—C2—C3	−141.1 (10)	C8—C9—C10—C11	−179.6 (7)
C2^i^—C1—C2—C3	38.9 (10)	C10^i^—C9—C10—C11	−59.6 (8)
N1—C1—C2—C3	−170.7 (7)	C9—C10—C11—C10^i^	59.9 (8)
C1^i^—C1—C2—C3^i^	−149.2 (8)	C10^i^—C10—C11—C12	120.0 (7)
C2^i^—C1—C2—C3^i^	30.8 (8)	C9—C10—C11—C12	179.9 (7)
N1—C1—C2—C3^i^	−178.9 (6)	C10^i^—C10—C11—C12^i^	84.3 (7)
C4—O2—C3—C3^i^	−96.3 (6)	C9—C10—C11—C12^i^	144.2 (7)
C4—O2—C3—C2	160.7 (11)	C10^i^—C11—C12—C12^i^	−81.1 (7)
C3^i^—O2—C3—C2	−102.9 (16)	C10—C11—C12—C12^i^	−120.6 (7)
C4—O2—C3—C2^i^	−147.2 (4)	C10^i^—C11—C12—C13	−141.5 (8)
C3^i^—O2—C3—C2^i^	−50.9 (9)	C10—C11—C12—C13	179.0 (7)
C1^i^—C2—C3—C3^i^	−50.3 (13)	C12^i^—C11—C12—C13	−60.4 (8)
O1—C2—C3—C3^i^	94.5 (6)	C11—C12—C13—C12^i^	60.7 (8)
C2^i^—C2—C3—C3^i^	0.000 (9)	C12^i^—C12—C13—C14	85.3 (10)
C1—C2—C3—C3^i^	−26.3 (6)	C11—C12—C13—C14	146.0 (10)
C1^i^—C2—C3—O2	33 (3)	C12^i^—C12—C13—C14^i^	118.0 (9)
O1—C2—C3—O2	178.0 (14)	C11—C12—C13—C14^i^	178.6 (9)
C2^i^—C2—C3—O2	83.5 (17)	C12^i^—C13—C14—C14^i^	118.9 (7)
C1—C2—C3—O2	57.2 (19)	C12—C13—C14—C14^i^	80.9 (8)
C3^i^—C2—C3—O2	83.5 (17)	C12^i^—C13—C14—C15	−177.5 (9)
C1^i^—C2—C3—C2^i^	−50.3 (13)	C12—C13—C14—C15	144.4 (9)
O1—C2—C3—C2^i^	94.5 (6)	C14^i^—C13—C14—C15	63.5 (12)
C1—C2—C3—C2^i^	−26.3 (6)	C14^i^—C14—C15—C16	−100.6 (6)
C3^i^—C2—C3—C2^i^	0.000 (15)	C13—C14—C15—C16	−164.2 (7)
C3^i^—O2—C4—C5	166.3 (13)	C13—C14—C15—C14^i^	−63.6 (12)
C3—O2—C4—C5	−166.3 (13)	C14^i^—C15—C16—C17	162.5 (9)
O2—C4—C5—C6	−163.4 (9)	C14—C15—C16—C17	−162.5 (9)
O2—C4—C5—C6^i^	163.4 (9)		

Symmetry codes: (i) *x*, −*y*+1/2, *z*.

### Hydrogen-bond geometry (Å, °)

**Table:**

*D*—H···*A*	*D*—H	H···*A*	*D*···*A*	*D*—H···*A*
O1—H1D···Br1	0.85	2.41	3.207 (6)	157.
C19—H19B···O1^ii^	0.96	2.23	3.190 (6)	176.

Symmetry codes: (ii) *x*+1, *y*, *z*.

## References

[bb1] Bruker (2007). *SMART* and *SAINT* Bruker AXS Inc., Madison, Wisconsin, USA.

[bb2] Fu, S., Wei, Z., Wei, X. & Wu, T. (2009). *Acta Cryst.* E**65**, o1836. 10.1107/S160053680902635X PMC2977172 21583537

[bb3] Koh, L. L., Xu, Y., Gan, L. M., Chew, C. H. & Lee, K. C. (1993). *Acta Cryst.* C**49**, 1032–1035.

[bb4] Liu, J., Wei, Z., Wei, X. & Zhang, C. (2010). *Acta Cryst.* E**66**, o2865. 10.1107/S1600536810040705 PMC3009037 21589047

[bb5] Sheldrick, G. M. (1996). *SADABS* University of Göttingen, Germany.

[bb6] Sheldrick, G. M. (2008). *Acta Cryst.* A**64**, 112–122. 10.1107/S0108767307043930 18156677

[bb7] Yin, B. L., Zhang, G. Y. & Wei, X. L. (1998). *China Surfact. Deterg. Cosmet.* **4**, 18–22.

